# A comparison of neoadjuvant therapies for gastroesophageal and gastric cancer on tumour resection rate: A network meta-analysis

**DOI:** 10.1371/journal.pone.0275186

**Published:** 2022-09-26

**Authors:** Seow Chee Herng, Norah Htet Htet, Cho Naing

**Affiliations:** 1 International Medical University, Kuala Lumpur, Malaysia; 2 University of New South Wales, Sydney, Australia; 3 Division of Tropical Health and Medicine, James Cook University, Townsville, Queensland, Australia; Fondazione IRCCS Istituto Nazionale dei Tumori, ITALY

## Abstract

**Background:**

Gastric cancer is one of the most common malignancies around the world, and a variety of neoadjuvant chemotherapies with different drug combinations are available for the treatment. R0 resection refers to a microscopically negative margin on resection, where no gross or microscopic tumour remains in the primary tumour. We aimed to find evidence on the relative effectiveness of neoadjuvant therapies for patients with advanced gastroesophageal and gastric cancer on the R0 resection rate.

**Methods:**

Relevant randomised controlled trials were searched using appropriate keywords in health-related databases. We performed network meta-analysis within a frequentist framework. The endpoint assessed was the R0 resection rate. We assessed consistency and transitivity assumptions that are necessary for network meta-analysis. This study only used data from published studies. The need for consent from participants was waived by the Ethics Review Committee of the International Medical University in Malaysia.

**Results:**

Six randomised controlled trials involving 1700 patients were identified. A network plot was formed with five neoadjuvant regimens [DLX (pyrimidine analogue + platinum compounds + chemoradiotherapy), DELX (pyrimidine analogue + epipodophylllotoxins/etoposide + platinum compounds + chemoradiotherapy), ADL (anthracycline + pyrimidine analogue + platinum compounds), ADM (anthracycline+ pyrimidine analogue + anti-folate compounds) and LTX (platinum compounds + taxane + chemoradiotherapy)] and surgery alone for management of patients with advanced gastroesophageal and gastric cancer. Assumptions required for a network meta-analysis such as consistency ((global test: *Chi*^2^ (1): 3.71; *p*:0.054)), and the transitivity in accord to the characteristics of interventions considered in this review were not violated. In the network comparison, surgery alone has a lower R0 resection rate compared with LTX (OR 0.2, 95%CI:0.01, 0.38) or DLX (OR 0.48, 95%CI: 0.29, 0.79). LTX has higher resection rate compared with DLX (OR 2.47, 95%CI: 1.08 to 5.63), DELX (OR 106.0, 95%CI: 25.29 to 444.21), ADM (OR 5.41, 95%CI: 1.56 to 18.78) or ADL (OR 3.12, 95%CI: 1.27 to 7.67). There were wide or very wide CIs in many of these comparisons. Overall certainty of the evidence was low or very low. Further research in this field is very likely to have an important impact on our confidence in the R0 resection rates between LTX versus other neoadjuvant chemotherapy is likely to change the estimate.

**Conclusions:**

Findings suggest that overall quality of evidence on the relative effectiveness of neoadjuvant chemotherapies was low to very low level. Therefore, we are very uncertain about the true effect of neoadjuvant therapies in the R0 resection rate in patients with gastroesophageal and gastric cancer. Future well-designed large trials are needed. To recruit large samples in this field, multicountry trials are recommended. Future trials also need to assess treatment-related adverse events, and patients-centered outcomes such as health‐related quality of life.

## Introduction

Gastric cancer is the fifth most frequently diagnosed cancer worldwide, and the third most common cause of cancer-related death [1, 2]. According to the WHO report, there were 782, 685 gastric cancer cases worldwide in 2018 [1, 3] with a majority of men [1, 4]. East Asian nations (e.g., Mongolia, Japan, and the Republic of Korea reported the high incidence rates. The Republic of Korea reported the highest incidence rate for both sexes [1], while Northern America, Northern Europe and Africa had lower rates [1].

Surgery is typically the primary intervention for the early stage of gastric cancer, and complete resection with adequate margin (> 4.0 cm) has been regarded as the standard goal [5]. Since most patients receive their diagnosis at the advanced stage, which is usually inoperable, early detection for gastric cancer is challenging. Moreover, the large-scale screening is impracticable for all countries, although endoscopic screening was recommended in Japanese Guideline for gastric Cancer Screening [6].

Various strategies including neoadjuvant chemotherapy has been used to improve the survival rate for patients with gastric cancer. The aim of the neoadjuvant chemotherapy is to reduce the tumour size [7] by the destruction of cancer cells [8] to improve the subsequent surgery outcome [7]. A poor response to neoadjuvant chemotherapy may delay curative surgery, and chemotherapy-induced toxicity may also increase the complications of surgery [9–11]. Different options of neoadjuvant chemotherapy was compared to surgery alone, or there are different regimens of neoadjuvant chemotherapy in various treatment protocols such as the Dutch Chemoradiotherapy for Oesophageal Cancer, followed by Surgery Study (CROSS) [12], European Organisation for Research and Treatment of Cancer (EORTC) [13], and 5-Fluorouracil, doxorubicin and methotrexate (FAMTX) [14].

A variety of neoadjuvant chemotherapy regimens with different drug combinations is available for the treatment of gastric cancer patients. For instance, different neoadjuvant chemotherapy regimens in terms of drug class were DLX (pyrimidine analogue + platinum compounds + chemoradiotherapy), DELX (pyrimidine analogue + epipodophyllotoxins/etoposide + platinum compounds + chemoradiotherapy), ADL (anthracycline pyrimidine analogue + platinum compounds), ADM (anthracycline + pyrimidine analogue + anti-folate compounds), and LTX (platinum compounds + taxane + chemoradiotherapy). Drug classes along with individual drug names are described in Table 1.

**Table 1:** Drug classes along with individual drug name.

Drug class	Drug name
Anthracycline (A)	Epirubicin, Doxorubicin
Pyrimidine analogue (D)	Fluorouracil (5-FU), Capecitabine (Cape)
Platinum compounds (L)	Cisplatin Oxaliplatin Carboplatin
Anti-folate agents (M)	Methotrexate
Epipodophyllotoxin (E)	Etoposide
Taxane (T)	Docetaxel
ACT (W)	Adjuvant chemotherapy
CRT (X)	Chemoradiotherapy

Hence, evidence on the relative effectiveness is imperative for selecting the optimum neoadjuvant chemotherapy. There were randomised controlled trials (RCTs) that investigated the efficacy and/or safety of various neoadjuvant chemotherapy regimens in treating gastric cancer. However, their relative effectiveness was unclear. There were a limited number of RCTs in this field probably because of logistic issues. As an example, RCT that directly compared LTX with DLX and reported the R0 reception rate is limited. R0 resection refers to a microscopically negative margin on resection, meaning that the primary tumour has been removed of gross or microscopic tumour [15]. The R0 resection rate has an impact on the treatment approach and the prognosis [16]. Although variations in the type of surgical resection depending on the type and site of the tumour (i.e., gastric cancer in this case), neoadjuvant chemotherapy could have improved the R0 resection rate [17].

An outcome of R0 resection rate is clinically significant because it provides the best chance for a cure by entirely removing any remaining cancer cells from the tumour bed [15]. It is one of the pathological assessment outcomes [12]. By removing the hidden micro metastases and down-staging the tumour, neoadjuvant chemotherapy regimens may improve the possibility of curative and complete resection during surgery [15]. As such, the appropriate neoadjuvant chemotherapy regimens can improve R0 resection rate and hence, it has a direct impact on the outcomes of surgery, and treatment success of gastric cancer.

We are aware of systematic reviews and meta-analyses that examined neoadjuvant chemotherapies in patients with gastric cancer (i.e., head-to-head comparison) [9, 18–20]. A pairwise meta-analysis is limited to the comparisons of two parallel therapies. A network meta-analysis includes direct and indirect comparisons (i.e., treatments are compared across RCTs using a common comparator treatment) and allows for inferences about the intervention effectiveness [21, 22]. Overall, the objective of the current study was to find the evidence on the relative effectiveness of neoadjuvant therapies for patients with advanced gastroesophageal and gastric cancer.

## Materials and methods

A protocol of this study was approved by the Ethics Review Committee of the International Medical University in Malaysia (ID: BMS 1/2019 (007)). We reported the current study in adherence to the Preferred Reporting Items for Systematic Reviews and Meta-Analysis (PRISMA-NMA) guideline [23] (S1 Checklist). This study only used data from published studies. The need for consent from participants was waived by the Ethics Review Committee of the International Medical University in Malaysia.

### Search strategy

We searched relevant studies in the health-related databases such as PubMed, Ovid MEDLINE, Ovid Embase, The Cochrane Library, and Google Scholar. The search strings were based on MeSH terms, including “gastric cancer” “neoadjuvant therapy” and “Surgery”. These terms were used in different combinations with no limitations to publication status. Study search was restricted to publications in the English language up to July 2021. A search strategy for PubMed is given in S1 Box. An additional search was done in ClinicalTrials.gov (http://www.clinicaltrials.gov/), WHO International Clinical Trials Registry Platform (http://apps.who.int/trialsearch/Default.aspx), EU Clinical Trials Register (https://www.clinicaltrialsregister.eu/). For any additional eligible studies, we checked the reference list of the relevant review and retrieved articles.

### Inclusion criteria

The inclusion criteria were framed based on the PICOS format [24].

**Study participants** (P): Patients diagnosed with gastroesophageal or gastric cancer, regardless of sex, age, anatomical site and tumour staging.

**Interventions** (I): Neoadjuvant chemotherapy, regardless of any drug combination

**Controls** (C): Alternative regimen of neoadjuvant chemotherapy, or surgery alone.

**Outcome** (O): The endpoint is the R0 resection rate.

R0 resection is as defined in the primary study. In general, it is a microscopically margin-negative resection, in which no gross or microscopic tumour remains in the primary tumour bed. In this study, the Ro resection rate was proportioned in two groups and measured as odds ratio (OR) and its 95% CI.

**Study design (S)**: Randomised controlled trial (RCT)

### Data extraction

One review author (SCH) screened the titles and abstracts of study search and retrieved the full-text of all potentially relevant articles. Another review author (NHH) double-checked this. Based on the inclusion criteria, the two review authors (SCH, NHH) independently assessed the full-text articles for eligibility. Using a piloted data extraction sheet, both investigators independently extracted data from the included studies. Data collected were author, publication year, country, participant’s characteristics, details of intervention and controls regimen (i.e., dosage, formulation, route of administration, duration), outcome measures, and follow-up time points of the outcome. For duplicates, we chose a publication that had the maximum data accessible. Any disagreements between the two investigators were settled by consensus.

### Methodological quality assessment

The Cochrane risk of bias tool was used to evaluate the methodological quality of the included RCTs [18]. We assessed the risk of bias in four domains such as random sequences generation, allocation concealment, blinding of participants and blinding of outcome assessors. For overall quality, we used the GRADE (Grading of Recommendations Assessment, Development, and Evaluation) [25].

### Statistical analysis

The endpoint used in the present study was a clinically important outcome such as the R0 resection rate.

#### Pairwise comparison

Direct pairwise meta-analysis was done with the use of standard frequentist approaches. For individual RCT, OR and corresponding 95% CI of the R0 resection rate was used to compare the groups. We preferred the intention-to-treat (ITT) analysis that included all randomized participants with outcome data. For pooling of the studies, summary OR and corresponding 95% CI was used.

We chose random effects model for pooling of studies so to allow that the true effect could vary from study to study [26]. We used the DerSimonian-Laird random-effects model [24]. To investigate heterogeneity, we used statistical tests such as p value from the *Chi*^2^ test and the *I*^2^ test. A p value of 0.10 was used to determine statistical significance in the *Chi*^2^ test. For the *I*^2^ test, 50% or more is regarded as substantial heterogeneity [27].

To investigate heterogeneity in a network of interventions, we used *tau*^2^, which suggests the presence of important heterogeneity (S2 Table). This approach address heterogeneity that cannot readily be explained by other factors [27].

#### Network meta-analyses

A network meta-analysis within a frequentist framework was done with random-effects models [28, 29]. A network connection was plotted. An assumption of network consistency was assessed as described elsewhere [30]. We assessed an assumption of consistency by design-by-treatment interaction model as this approach could allow for a global test for the presence of inconsistency [31]. ‘Global approaches’ evaluate coherence in the entire network [32]. As such, the χ^2^-test was used to estimate the statistical significance of all possible inconsistencies in the networks [33].

We evaluated the plausibility of the network meta-analysis transitivity assumption by looking at inclusion and exclusion criteria of included studies (i.e., PICOS in this case), and checked the characteristics of the included RCTs [34]. The transitivity assumption means that participants included in different trials with different treatments (for gastroesophageal and gastric cancer in this case) can be considered as part of a multiarmed RCT, and could potentially have been randomised to any of the interventions [22].

The network meta-analysis results were reported for ‘mixed treatment contrasts’, including both direct and indirect evidence from across the entire network [29]. For a ranking of the effectiveness, we reported probability values as ‘Surface Under the Cumulative Ranking Curve’ (SUCRA) [29]. SUCRA = 1 or 0 reflects whether an intervention ranked first or last. All analysis was done with STATA 15.0 (Stata Corp, TX).

#### Assessing the overall quality of evidence

We assessed the overall quality of evidence derived from the pairwise and network meta-analysis, following the GRADE approach [28, 32, 35–38]. As described elsewhere [32], the quality rating on GRADE approach can be classified as ‘no limitation’ (not important enough to warrant downgrading), ‘serious’ (downgrading the certainty of rating by one level) or ‘very serious’ (downgrading the certainty of rating by two levels) for the following five domains.

■ Risk of bias (e.g., lack of allocation sequence concealment, lack of blinding)
■ Inconsistency of results (i.e., widely differing estimates of effect)
■ Indirectness of evidence (e.g., surrogate outcome when data on patient-important outcomes are not available)
■ Imprecision of results (i.e., wide 95% CIs including null effect)
■ High probability of publication bias.

## Results

**Fig 1:**
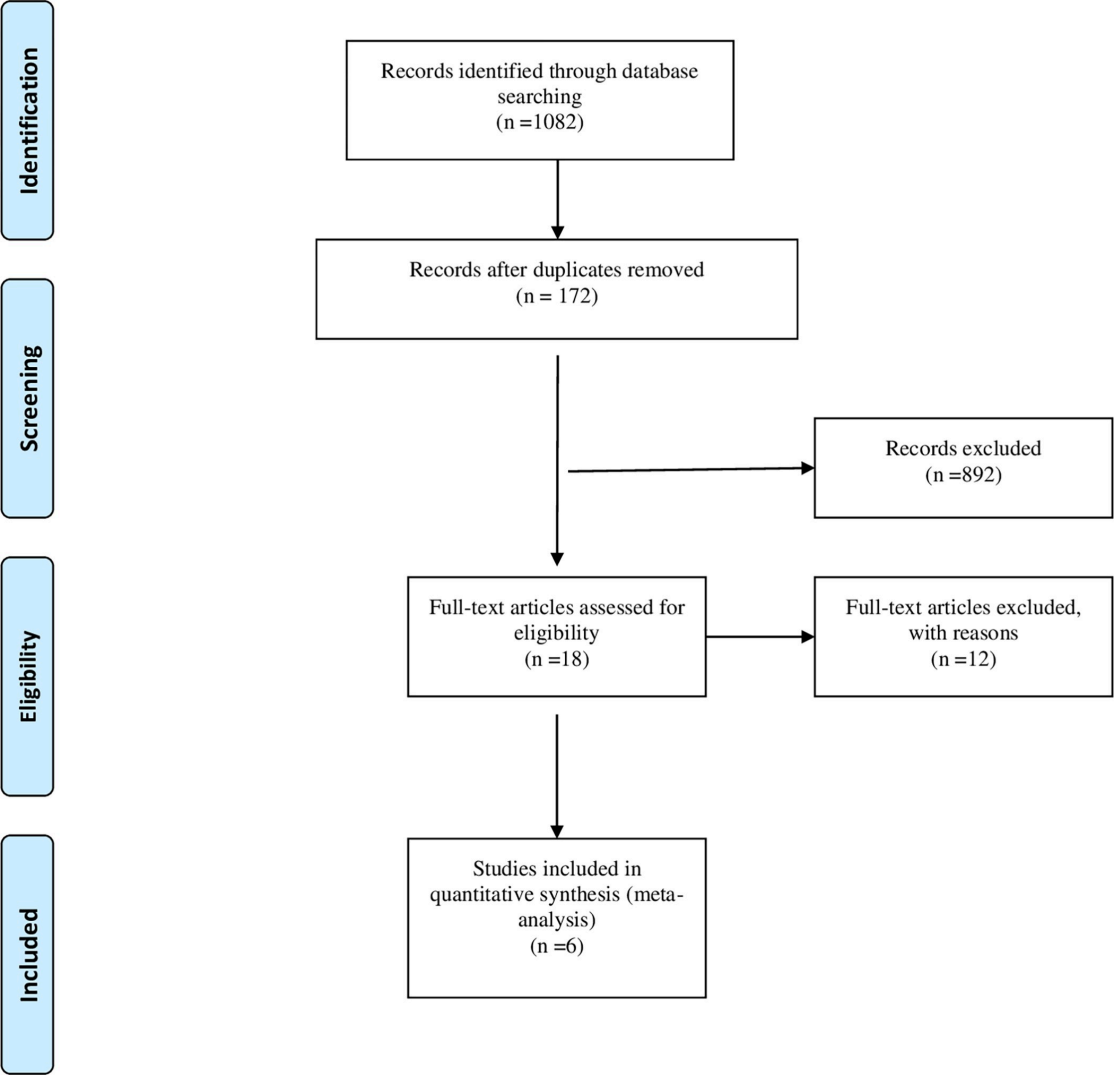
Study selection PRISMA flowchart.

Fig 1. shows the study selection process. The initial search yielded 1082 citations, and after the screening of title and abstract, 18 full-text papers were reviewed, and a final of six studies (n = 1700) was included in this review [12–14, 39–41]. The studies identified were published between 2004 and 2018. A summary of 12 excluded studies is provided in S1 Table.

The characteristics of the six included RCTs are provided in Table 2. The number of participants ranged from 59 [24] to 788 [39]. Two studies were conducted in France [13, 41], or Netherlands [12, 14], one study in Germany [40], and another single study was a multicounty study in Netherland, Sweden and Denmark [39]. Six treatment options, including surgery alone were examined in this review. All studies identified were two-arm trials. The most frequent comparison was DLX versus surgery alone in two studies [13, 41]. Information on individual drug names and their drug classes used in the regimen are provided in Table 3.

**Table 2:** Characteristics of the included studies.

Study, Yr [ref #]	Country	Study period	Study design	Total	M%	Mean age (age range) in yr	Type of cancer	Cancer stage	Interventions/ control (n) #	Trial name
Cats, 2018 [39]	Netherland, Sweden, Denmark	1/2007–4/2015	RCT phase III	788	67%	54–69	GC	R	ADL vs DLX	CRITICS
van Hagen 2012 [12]	Netherland	3/2004–12/2008	multicentre, RCT phase III	366	75%/81%	60 (36–79) 60 (36–73)	EC, GEJ	NA	LTX vs SUR	CROSS
Schuhmacher, 2010 [13]	France	7/1999–2/2004	RCT	144	69.4%/ 69.4%	38–70 26–69	GC, GEJ	locally advanced	DLX vs SUR	EORTC
Stahl, 2017 [40]	Germany		prospective RCT phase III trial	119	NA	NA	GEJ	locally advanced	DLX vs DELX	POET
Hartgrink, 2004 [14]	Netherland	9/1993–1/1996	prospective RCT, multicentre	59	NA	NA	GC	R	ADM vs SUR	FAMTX
Ychou, 2011 [41]	France	11/1995–12/2003	multicenter RCT	224	85% /82%	63 (36–75) 63 (38–75)	GC, GEJ, EC	R	DLX vs SUR	FNCLCC and FFCD

* Cancer stage at the time of the study

#: please see details in Tables 1 and 3; GC: gastric cancer; GEJ: gastroesophageal junction; EC: oesophageal cancer; R: resectable

RCT: randomised controlled trial; SUR: surgery; Yr: year of publication

ADL: anthracycline + pyrimidine analogue + platinum; ADM: anthracycline + pyrimidine analogue + folic acid analogue

DLX: pyrimidine analogue + platinum compounds + radiotherapy; DELX: pyrimidine analogue + Epipodophyllotoxins + platinum compounds + radiotherapy

CROSS: the Dutch Chemoradiotherapy for Oesophageal Cancer followed by Surgery Study; EORTC: European Organisation for Research and Treatment of Cancer

FAMTX: 5-Fluorouracil, doxorubicin and methotrexate

POET: PreOperative therapy in Esophagogastric adenocarcinoma Trial

FNCLCC: the Federation Nationale des Centres de Lutte contre le Cancer

FFCD: the Federation Francophone de Cancerologie Digestive

**Table 3:** Drug name of the drug class in the included trials.

Study, Yr [ref #]	Intervention (drug names)	Control (drug names)
Cats, 2018 [39]	ADL Epirubicin + cisplatin/oxaliplatin + capecitabine (chemotherapy)	DLX (Capecitabine + cisplatin + radiotherapy) 45Gy in 25 fractions of 1.8 Gy (chemoradiotherapy)
Van Hagen, 2012 [12]	LTX Carboplatin + paclitaxel + 41.4 Gy in 23 fractions + (chemoradiotherapy)	Surgery alone
Schuhmacher,2010 [13]	DLX Cisplatin + folinic acid + fluorouracil	Surgery alone
Hartgrink, 2004 [14]	ADM df (FAMTX regimen) 5-FU + doxorubicin + methotrexate	Surgery alone
Stahl, 2017 [40]	DLX 5-FU + cisplatin (chemotherapy)	DELX Chemoradiotherapy + surgery 5-FU + cisplatin + etopoxide + 30 Gy 15 fractions of 2Gy
Ychou, 2011 [41]	DLX 5-FU + cisplatin	Surgery alone

### Methodological quality assessment

A summary of the risk of bias assessment of the included studies is presented in S1 Fig. Majority were ‘unclear’ risk of bias related to random sequences generation of participant selection, blinding of participants, and outcome assessment.

### Pairwise meta-analysis for the R0 resection rate

A total of six RCTs (n = 1700) were identified [12–14, 39–41]. Two RCTs compared different neoadjuvant chemotherapy regimens with post-op adjuvant chemoradiotherapy [39, 40]. Four RCTs compared different regimens of NAC with surgery alone [12–14, 41].

Generally, in the direct head-to-head comparison, neoadjuvant chemotherapy had a higher R0 resection rate than surgery alone. For more details, DLX had a twofold increase in the R0 resection rate than surgery alone (OR 2.08; 95% CI 1.26–3.43; *Tau*^2^ = 0.00; *Chi*^2^ = 0.02, *df* = 1,P = 0.88; *I*^2^: 0% 368 participants; 2 studies).

**Fig 2:**
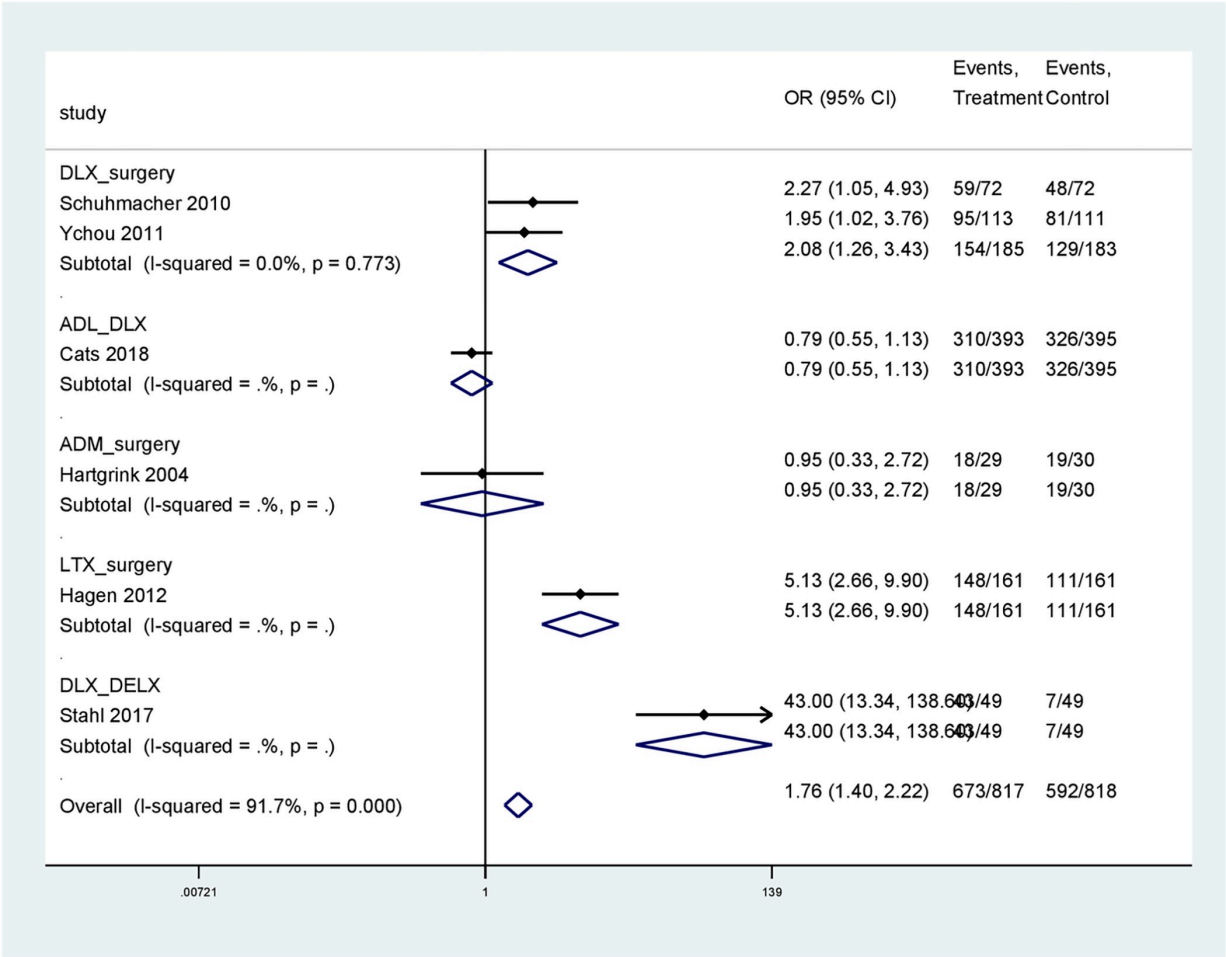
Pairwise meta-analysis for the Ro resection rate.

LTX had a five-fold increase in the R0 resection rate of gastroesophageal and gastric cancer than surgery alone (OR 5.13; 95% CI 2.66–9.9; 322 participants; 1 study). DLX had a higher Ro resection rate than DELX (OR 43.00; 95% CI 13.4–138.6; 98 participants; 1 study). Of note is very wide CI. ADL and DLX had comparable R0 resection rates (OR 0.79; 95% CI 0.55–1.13; 788 participants; 1 study (Fig 2).

### Comparative efficacy

**Fig 3:**
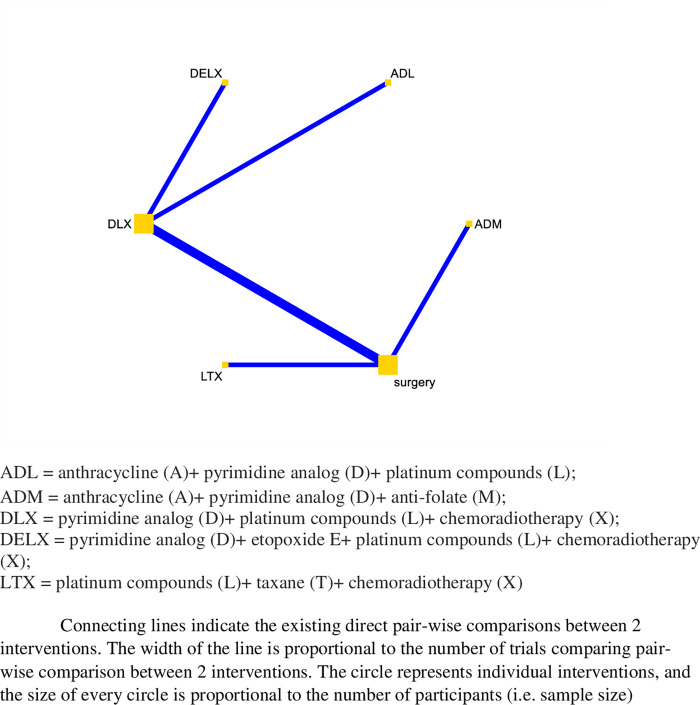
R0 resection network plot.

Fig 3 shows a network plot of five different neoadjuvant chemotherapy regimens (i.e., ADL, ADM, DELX, DLX, LTX), and surgery alone for the management of patients with advanced gastric cancer.

**Fig 4:**
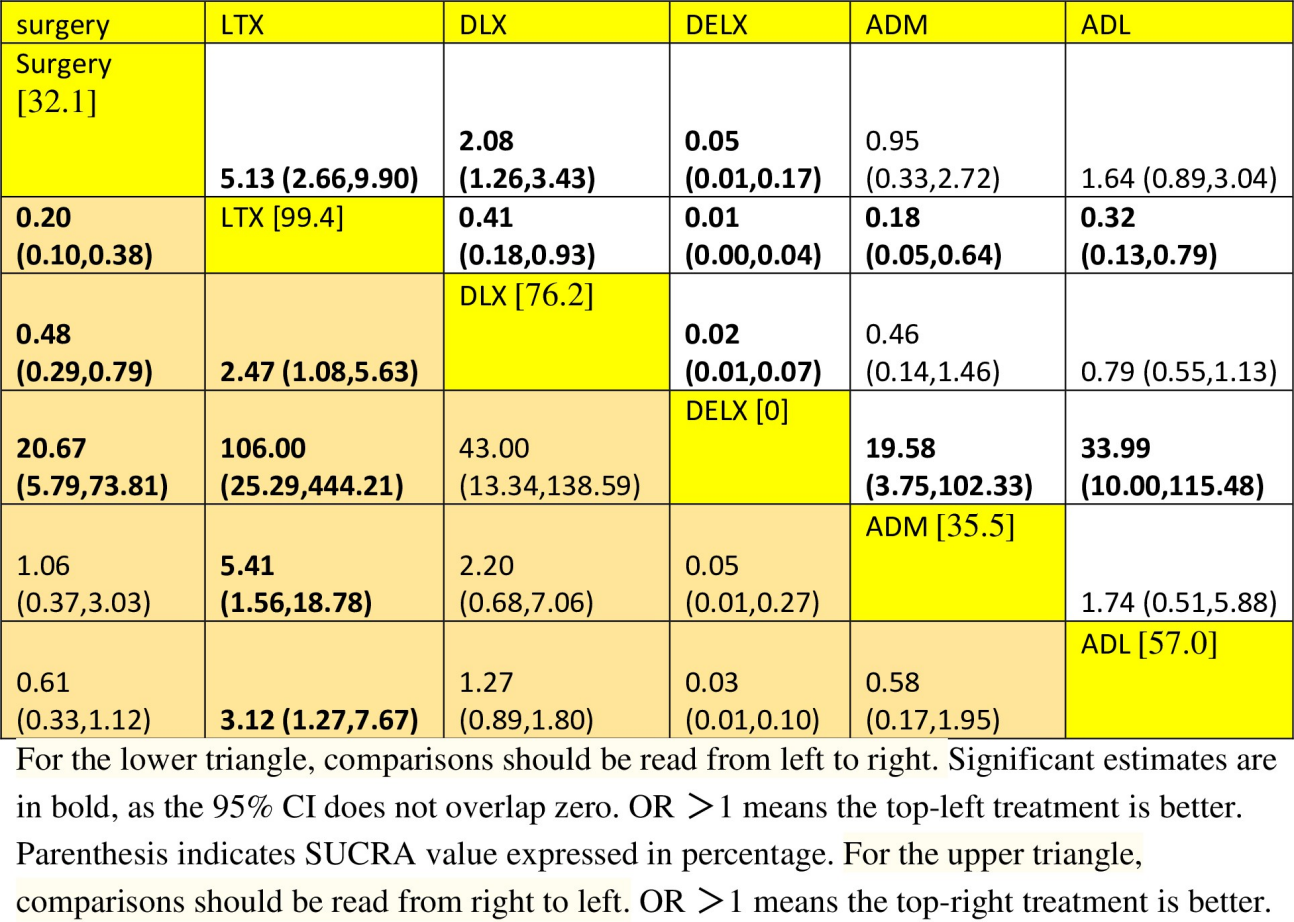
Net league showing the relative Ro resection rate. For the lower triangle, comparisons should be read from left to right. Significant estimates are in bold, as the 95% CI does not overlap zero. OR > 1 means the top-left treatment in better. Parenthesis indicates SUCRA value expressed in percentage. For the upper triangle, comparisons should be read from right to left. OR > 1 means the top-right treatment in better.

**Fig 5:**
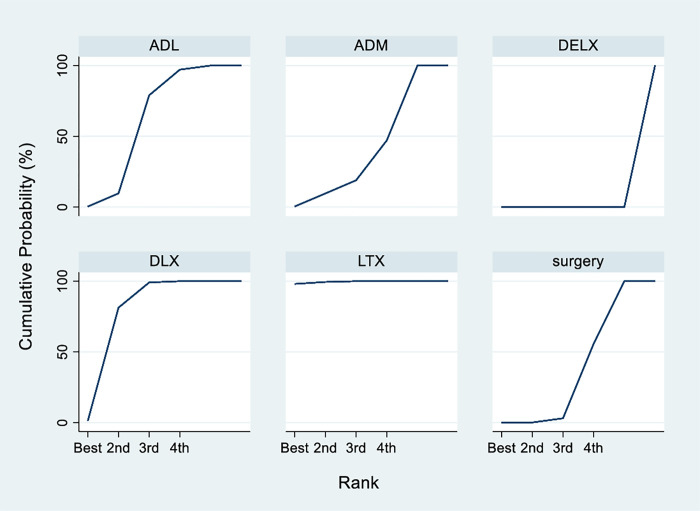
Plot showing the assessment of SUCRA.

In the network comparison, surgery alone has a lower R0 resection rate compared with LTX (OR 0.2, 95%CI: 0.01, 0.38), or DLX (OR 0.48, 95%CI: 0.29, 0.79). LTX has higher resection rate compared with DLX (OR 2.47, 95%CI: 1.08 to 5.63), DELX (OR 106.0, 95%CI: 25.29 to 444.21), ADM (OR 5.41, 95%CI: 1.56 to 18.78), or ADL (OR 3.12, 95%CI: 1.27 to 7.67). Of note, there were wide or very wide CIs in many of these comparisons (Fig 4). Based on the current analysis, LTX is being the best neoadjuvant chemotherapy for R0 resection rate, holding the highest SUCRA value (99.4%), followed by DLX (76.2%) (Fig 5).

The forest plot (S2 Fig) shows the individual study results, grouped by treatment contrast and design. The similarity of pooled within the designs and pooled overall results support the consistency model [22]. In this network meta-analysis, an assumption of consistency was not violated according to global approaches (*Chi*^2^: 3.71, p:0.054) (S2 Fig). Because of the inclusion criteria (i.e., PICOS), and the characteristics of interventions considered in this review, we had no obvious concerns about the transitivity assumption [34], although we cannot rule this out completely.

### Overall certainty of evidence

Overall, LTX was better efficacy in terms of R0 resection rate than DLX (low certainty of evidence), DELX (very low certainty of the evidence), ADM (very low certainty of evidence), and ADL (very low certainty of the evidence) or surgery alone (low certainty of the evidence) as neoadjuvant chemotherapy for patients with gastric cancer (Table 4). Described elsewhere [24], this implied that further research is very likely to have an important impact on our confidence in the estimated effects between LTX versus other neoadjuvant chemotherapy.

**Table 4:** GRADE quality of evidence for the comparative R0 resection rate.

Comparison	Direct evidence		Indirect evidence		Network meta-analysis	
	OR (95%CI)	Quality of evidence	OR (95%CI)	Quality of evidence	OR (95%CI)	Quality of evidence
LTX vs surgery	5.13 (2.66–9.90	⨁⨁◯◯ low^a,^^b^^,c^	5.13 (2.66–9.90	⨁⨁◯◯ low^a,^^d^	5.13 (2.66,9.90)	⨁⨁◯◯ low^a,^^b^^,c^
LT X vs DLX	-		2.47 (1.08,5.63)	⨁◯◯◯ Very low^a,^^b^^,^^d^	2.47 (1.08,5.63)	⨁◯◯◯ Very low^a,^^b^^,c^
LT X vs DELX	-		106.00 (25.29,444.21)	⨁◯◯◯ Very low^a,^^b^^,^^d^	106.00 (25.29,444.21)	⨁◯◯◯ Very low^a,^^b^^,c^
LT X vs ADL	-		3.12 (1.27,7.67)	⨁◯◯◯ Very low^a,^^b^^,^^d^	3.12 (1.27,7.67)	⨁◯◯◯ Very low^a,^^b^^,c^
LT X vs ADM	-		5.41 (1.56,18.78)	⨁◯◯◯ Very low^a,^^b^^,^^d^	5.41 (1.56,18.78)	⨁◯◯◯ Very low^a,^^b^^,c^
LTX vs DELX	-		106.00 (25.29,444.21)	⨁◯◯◯ Very low^a,^^b^^,^^d^	106.00 (25.29,444.21)	⨁◯◯◯ Very low^a,^^b^^,^^d^

^l^Limitations (risk of bias).

^b^Inconsistency.

^i^Imprecision.

^d^ Severe imprecision

GRADE Working Group grades of evidence

**High certainty:** we are very confident that the true effect lies close to that of the estimate of the effect.

**Moderate certainty:** we are moderately confident in the effect estimate: the true effect is likely to be close to the estimate of the effect, but there is a possibility that it is substantially different.

**Low certainty:** our confidence in the effect estimate is limited: the true effect may be substantially different from the estimate of the effect.

**Very low certainty:** we have very little confidence in the effect estimate: the true effect is likely to be substantially different from the estimate of effect.

## Discussion

The present network meta-analysis identified six RCTs, involving 1700 participants with gastroesophageal and gastric cancer across five European countries. Major findings are as described below.

1. Characteristics of the study population were comparable in the trials included.
2. Neoadjuvant chemotherapy regimens of the included trials were standardized. Chemotherapy drugs class included anthracycline, pyrimidine analogue, platinum compounds, folic acid analogue, taxane and epipodophyllotoxins. The most frequently used drug class in the current study is pyrimidine analogue + platinum compounds + radiotherapy (DLX).
3. Most of the studies included are unclear risk of bias as per selection bias (random sequence generation), performance bias (blinding of participants and individual), detection bias (blinding of outcome assessment) and low risk of bias in selection bias (allocation concealment).
4. For the endpoint assessment of R0 resection rate, this network meta-analysis showed LTX (a combination of chemoradiotherapy, platinum compounds and taxane) was the best-favoured neoadjuvant chemotherapy regimen for management of gastroesophageal and gastric cancer patients compared to alternative neoadjuvant chemotherapy, or surgery alone.
5. Overall quality of evidence was low to very low. Therefore, we have very little confidence in the effect estimate: the true effect is likely to be substantially different from the estimate of effect.

The most important question regarding the neoadjuvant chemotherapy for gastroesophageal or gastric cancer is which is the best regimen. We are aware that the regimen of the neoadjuvant chemotherapy might have an impact on the outcome of the treatment. Therefore, the regimen specific factors concerning neoadjuvant chemotherapy for gastroesophageal or gastric cancer should take into account [9]. According to the CRITICS trial, the chemoradiotherapy group (DLX) has a higher resection rate than the chemotherapy group (ADL) [39]. It can be assumed that the combination of chemotherapy and radiotherapy could improve the R0 resection rate and has a superior surgical outcome. However, in the present analysis, we did not consider the dosage, duration of chemotherapy regimens, type, site, and stage of the tumour at the time of diagnosis and treatment. Our findings could be, to a certain extent, explained in the pharmacological context. For instance, etoposide (Triptolide)-based regimen (DELX in this case) could induce tumour cell apoptosis directly and it also enhanced apoptosis through cytotoxic agents such as TNF-α [42].

A published review with head-head comparison reported that the R0 resection rate of gastroesophageal or gastric cancers was higher in the treatment group (i.e., neoadjuvant chemotherapy in this study) than in the control group (OR 1.38, 95%CI 1.03–1.85, 4 studies) [9]. This was supporting the findings in the current pairwise analysis as well as the network meta-analysis. But, the overall quality of evidence was low or very low. Hence, further research is very likely to have an important impact on our confidence in the estimate of effect between neoadjuvant chemotherapy regimens (i.e., LTX, DLX) versus alternative neoadjuvant chemotherapy regimens (ADL, ADL, DELX), or surgery alone.

### Study limitations

We acknowledged some limitations in the present study. This study was addressed based on drug class due to complexity and variation in regimens (dose, route, and duration). For instance, anthracycline, pyrimidine analogue, platinum compounds, and taxane are different drug classes. Moreover, subgroup analysis with staging or anatomical sites of gastric cancer were not done due to the limited number of studies. Only a few RCTs assessed this clinically important outcome. Thus, the comprehensiveness of interpretation may be limited. Moreover, some studies included were with a high risk of bias in performance bias and detection bias domains. This could have an impact on the outcome estimates. Most of the individual RCTs in the current analysis were small studies and underpowered to identify the important differences in effect estimates. Additionally, there was a geographical imbalance of the trials due to an absence of studies from the Asian region pertinent to the Republic of Korea, which has the highest incidence of gastric cancer.

### Implications for practice

Our review includes direct and indirect comparisons that showed the role of neoadjuvant chemotherapy in the R0 resection rate in patients with gastroesophageal and gastric cancer. On the basis of low to very low-quality evidence, we have very little confidence in the effect estimate and the true effect of neoadjuvant therapies is likely to be substantially different from the effect estimate. There is presently insufficient high-quality data for a definitive statement on whether differing neoadjuvant regimens differ from each other in their effects on R0 resection rate.

### Implications for research

Well-designed large RCTs of neoadjuvant therapies on patients-centered outcomes (e.g. health‐related quality of life, serious adverse events) that are considered most relevant to patients and healthcare services, should be undertaken using consistent methods for reporting and considering of clinically important benefits of these drugs. In order to get a sufficient number of participants, multicenter/multicountry trials are recommended.

## Conclusions

Findings suggest that the overall quality of evidence on the relative effectiveness of neoadjuvant therapies in the R0 resection rate is of low to very low. Therefore, we are very uncertain about the true effect of neoadjuvant therapies in the R0 resection rate in patients with gastroesophageal and gastric cancer. Future well-designed large trials are needed. To recruit large samples in this field, multicountry trials are recommended. Future trials also need to assess treatment-related adverse events, and patients-centered outcomes such as health‐related quality of life.

## Supporting information

S1 Checklist PRISMA-NMA checklist. (DOC)

S1 Box Search strategies in PubMed. (DOC)

S1 Table Summary of the excluded studies. (DOC)

S2 Table Direct and indirect estimates. (DOC)

S1 Fig Methodological quality assessment. (PDF)

S2 Fig Forest plot for neoadjuvant therapies. (PDF)
