# Supplementary material for: A comparison of neoadjuvant therapies for gastroesophageal and gastric cancer on tumour resection rate: A network meta-analysis
Source: PLoS One. 2022 Sep 26;17(9):e0275186. doi: 10.1371/journal.pone.0275186 (PMC9512180; doi:10.1371/journal.pone.0275186)
Supplement: S1 Box — (DOC) [file pone.0275186.s002.doc]

**S1 Box. Search strategies in PUBMED**

| (("stomach neoplasms"[MeSH Terms] OR ("stomach"[All Fields] AND "neoplasms"[All Fields]) OR "stomach neoplasms"[All Fields] OR ("gastric"[All Fields] AND "cancer"[All Fields]) OR "gastric cancer"[All Fields]) AND ("neoadjuvant therapy"[MeSH Terms] OR ("neoadjuvant"[All Fields] AND "therapy"[All Fields]) OR "neoadjuvant therapy"[All Fields])) AND ("randomized controlled trial"[All Fields] OR "randomized controlled trials as topic"[MeSH Terms] OR "randomised clinical trial"[All Fields] OR "randomized clinical trial"[All Fields]) AND ("humans"[MeSH Terms] OR "humans"[All Fields] OR "human"[All Fields]) |
| --- |
