# Supplementary material for: A comparison of neoadjuvant therapies for gastroesophageal and gastric cancer on tumour resection rate: A network meta-analysis
Source: PLoS One. 2022 Sep 26;17(9):e0275186. doi: 10.1371/journal.pone.0275186 (PMC9512180; doi:10.1371/journal.pone.0275186)
Supplement: S1 Table — (DOC) [file pone.0275186.s003.doc]

S1 Table. Summary of the excluded studies

| Study | Study year | Reason of exclusion | Reference |
| --- | --- | --- | --- |
| Boige | 2007 | Only abstract | Boige V, et al. Final results of a randomized trial comparing preoperative 5-fluorouracil (F)/cisplatin (P) to surgery alone in adenocarcinoma of stomach and lower esophagus (ASLE): FNLCC ACCORD07-FFCD 9703 trial. JCO 2007 06/20; 2020/07;25(18):4510-4510. |
| Feng | 2015 | Not RCT (Retrospective study) | Feng D, et al Surgical outcomes in patients with locally advanced gastric cancer treated with S-1 and oxaliplatin as neoadjuvant chemotherapy. World journal of surgical oncology 2015 01/30;13:11-11. |
| Heger | 2014 | Not RCT (retrospective exploratory study) | Heger U, et al. Is preoperative chemotherapy followed by surgery the appropriate treatment for signet ring cell containing adenocarcinomas of the esophagogastric junction and stomach? Ann Surg Oncol 2014;21(5):1739-1748. |
| Imano | 2010 | Different outcome measured (apoptotic index) | Imano M et al. Prospective randomized trial of short-term neoadjuvant chemotherapy for advanced gastric cancer. European Journal of Surgical Oncology 2010 10/01; 2020/07;36(10):963-968. |
| Lorenzen | 2013 | Not RCT (pooled data from three multicentre phase II studies) | Lorenzen S et al. Impact of pathologic complete response on disease-free survival in patients with esophagogastric adenocarcinoma receiving preoperative docetaxel-based chemotherapy. Ann Oncol 2013;24(8):2068-2073. |
| Peixoto | 2014 | Not RCT (cohort study, from multicentre databases) | Peixoto RD et al. Perioperative chemotherapy for gastroesophageal cancer in British Columbia: a multicentre experience. Current oncology (Toronto, Ont.) 2014 04;21(2):77-83. |
| Slagter | 2018 | Ongoing study (multicentre RCT phase II non-comparative study) | Slagter AE et al. CRITICS-II: a multicentre randomised phase II trial of neo-adjuvant chemotherapy followed by surgery versus neo-adjuvant chemotherapy and subsequent chemoradiotherapy followed by surgery versus neo-adjuvant chemoradiotherapy followed by surgery in resectable gastric cancer. BMC Cancer 2018 09/10;18(1):877-877. |
| Sun | 2014 | Not RCT (retrospective cohort study) | Sun Z, et al Neoadjuvant chemotherapy with FOLFOX4 regimen to treat advanced gastric cancer improves survival without increasing adverse events: a retrospective cohort study from a Chinese center. The Scientific World Journal 2014;2014:418694-418694. |
| Wagner | 2019 | Ongoing (phase II trial) | Wagner AD et al. EORTC-1203-GITCG - the "INNOVATION"-trial: Effect of chemotherapy alone versus chemotherapy plus trastuzumab, versus chemotherapy plus trastuzumab plus pertuzumab, in the perioperative treatment of HER2 positive, gastric and gastroesophageal junction adenocarcinoma on pathologic response rate: a randomized phase II-intergroup trial of the EORTC-Gastrointestinal Tract Cancer Group, Korean Cancer Study Group and Dutch Upper GI-Cancer group. BMC Cancer 2019 05/24;19(1):494-494. |
| Yonemura | 2016 | Different outcome measured (peritoneal cancer index) | Yonemura Y et al. A comprehensive treatment for peritoneal metastases from gastric cancer with curative intent. European Journal of Surgical Oncology 2016 08/01; 2020/07;42(8):1123-1131. |
| Zhang | 2004 | Not RCT (Retrospective) | Zhang C et al. Clinical significance of preoperative regional intra-arterial infusion chemotherapy for advanced gastric cancer. World journal of gastroenterology 2004 10/15;10(20):3070-3072. |
| Zhang | 2012 | Retrospective record review | Zhang J et al. Efficacy and safety of neoadjuvant chemotherapy with modified FOLFOX7 regimen on the treatment of advanced gastric cancer. Chin Med J 2012;125(12). |
