# Supplementary material for: A comparison of neoadjuvant therapies for gastroesophageal and gastric cancer on tumour resection rate: A network meta-analysis
Source: PLoS One. 2022 Sep 26;17(9):e0275186. doi: 10.1371/journal.pone.0275186 (PMC9512180; doi:10.1371/journal.pone.0275186)
Supplement: S2 Table — (DOC) [file pone.0275186.s004.doc]

**S2 Table. Direct and indirect estimates**

**A : ADL; B: ADM; C: DELX; D: DLX; E: LTX; F: SUR**
