# Supplementary material for: A comparison of neoadjuvant therapies for gastroesophageal and gastric cancer on tumour resection rate: A network meta-analysis
Source: PLoS One. 2022 Sep 26;17(9):e0275186. doi: 10.1371/journal.pone.0275186 (PMC9512180; doi:10.1371/journal.pone.0275186)

S1 Fig 1

A) Assessment of the individual studies

Study, year	Randomization	Allocation concealment	Blinding of participants and individual	Blinding of outcome assessment
Cats 2018	Low	Low	High	Unsure
Cunningham 2006	Low	Low	Unsure	Unsure
Hartgrink 2004	Unsure	Low	Unsure	Unsure
Stahl 2017	Unsure	Low	High	High
Schuhmacher 2010	Unsure	Low	Unsure	unsure
Ychou 2011	Unsure	Low	Unsure	Unsure

LOW: low risk of bias; High: high risk of bias; Unsure: unsure riski of bias

B) Summary of the methodological quality assessment across 12 included studies

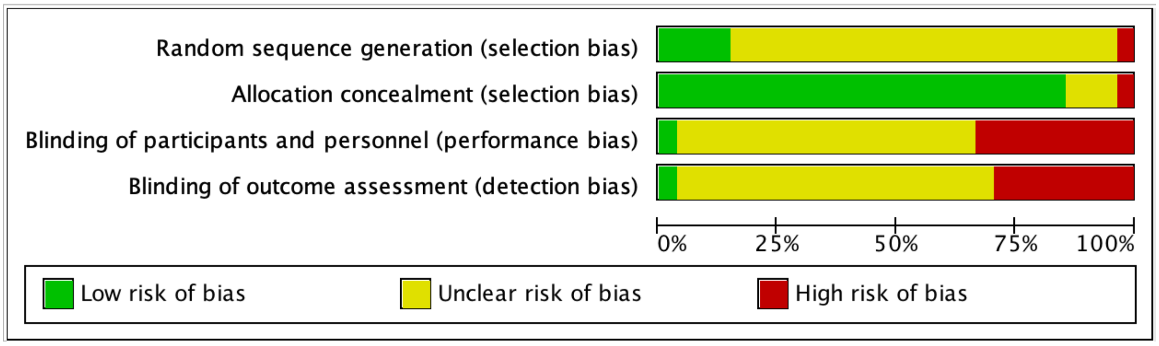

Supplement: S1 Fig — (PDF) [file pone.0275186.s005.pdf]
