# Supplementary figures and images for: A comparison of neoadjuvant therapies for gastroesophageal and gastric cancer on tumour resection rate: A network meta-analysis
Source: PLoS One. 2022 Sep 26;17(9):e0275186. doi: 10.1371/journal.pone.0275186 (PMC9512180; doi:10.1371/journal.pone.0275186)

**S2 Fig. Forest plot for NACs**

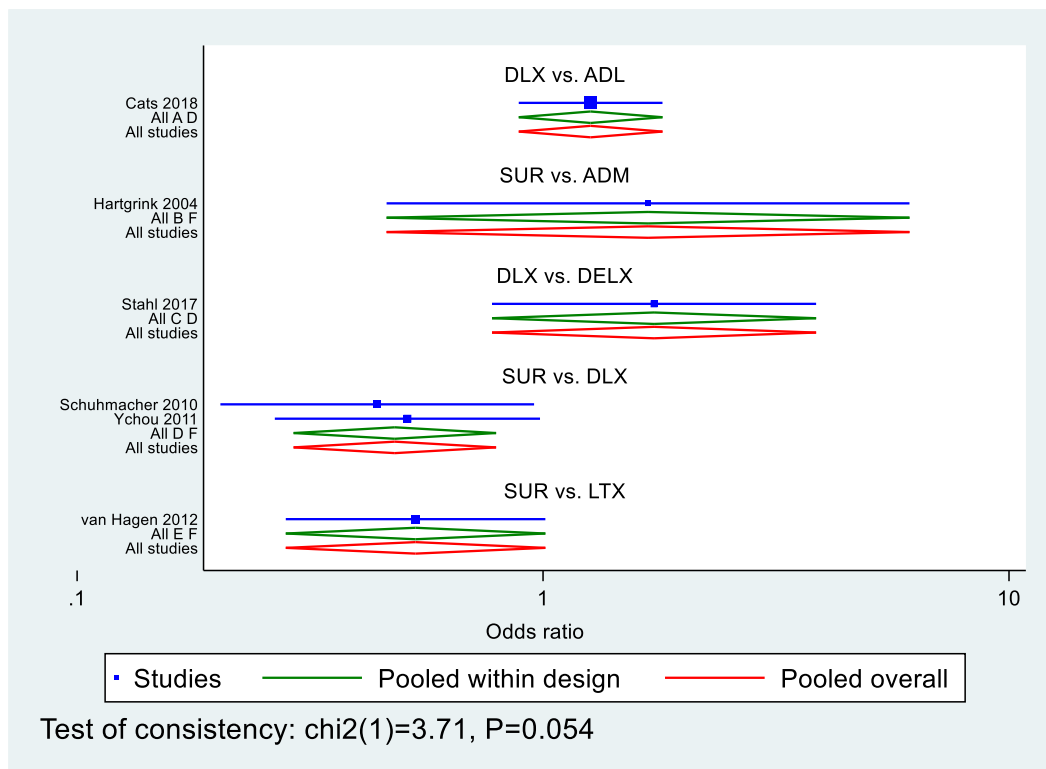

Supplement: S2 Fig — (PDF) [file pone.0275186.s006.pdf]
